# Unravelling the Influence of Chlorogenic Acid on the Antioxidant Phytochemistry of Avocado (*Persea americana* Mill.) Fruit Peel

**DOI:** 10.3390/antiox13040456

**Published:** 2024-04-12

**Authors:** Gloria O. Izu, Emmanuel Mfotie Njoya, Gaetan T. Tabakam, Jennifer Nambooze, Kgalaletso P. Otukile, Seiso E. Tsoeu, Victoria O. Fasiku, Ayodeji M. Adegoke, Ochuko L. Erukainure, Samson S. Mashele, Tshepiso J. Makhafola, Mamello P. Sekhoacha, Chika I. Chukwuma

**Affiliations:** 1 Centre for Quality of Health and Living (CQHL), Faculty of Health and Environmental Sciences, Central University of Technology, Bloemfontein 9301, South Africa; 221039621@stud.cut.ac.za (G.O.I.); enjoya@cut.ac.za (E.M.N.); tgaetan@cut.ac.za (G.T.T.); smashele@cut.ac.za (S.S.M.); jmakhafola@cut.ac.za (T.J.M.); 2 Department of Chemistry, Faculty of Natural and Agricultural Sciences, University of the Free State, Bloemfontein 9301, South Africa; 2020280521@ufs4life.ac.za (J.N.); 2020930810@ufs4life.ac.za (K.P.O.); 3 Molecular Sciences Institute, School of Chemistry, University of the Witwatersrand, Private Bag 3, Wits 2050, South Africa; 557872@students.wits.ac.za; 4 Department of Pharmacology, Faculty of Health Sciences, University of the Free State, Bloemfontein 9301, South Africa; fasiku.v@ufs.ac.za (V.O.F.); adegoke.am@ufs.ac.za (A.M.A.);; 5 Laser Research Centre, Faculty of Health Sciences, University of Johannesburg, Doornfontein 2028, South Africa

**Keywords:** avocado peel, chlorogenic acid, antioxidant, chromatography

## Abstract

Oxidative stress is pivotal in the pathology of many diseases. This study investigated the antioxidant phytochemistry of avocado (*Persea americana* Mill.) peel. Different solvent extracts (dichloromethane, ethyl acetate, methanol, and water) of avocado peel were subjected to total phenol and flavonoid quantification, as well as in vitro radical scavenging and ferric reducing evaluation. The methanol extract was subjected to gradient column chromatographic fractionation. Fraction 8 (eluted with hexane:chloroform:methanol volume ratio of 3:6.5:0.5, respectively) was subjected to LC-MS analysis. It was assessed for cellular inhibition of lipid peroxidation and lipopolysaccharide (LPS)-induced ROS and NO production. The DPPH radical scavenging mechanism of chlorogenic acid was investigated using Density Functional Theory (DFT). The methanol extract and fraction 8 had the highest phenol content and radical scavenging activity. Chlorogenic acid (103.5 mg/mL) and 1-*O*-caffeoylquinic acid (102.3 mg/mL) were the most abundant phenolics in the fraction. Fraction 8 and chlorogenic acid dose-dependently inhibited in vitro (IC_50_ = 5.73 and 6.17 µg/mL) and cellular (IC_50_ = 15.9 and 9.34 µg/mL) FeSO_4_-induced lipid peroxidation, as well as LPS-induced ROS (IC_50_ = 39.6 and 28.2 µg/mL) and NO (IC_50_ = 63.5 and 107 µg/mL) production, while modulating antioxidant enzyme activity. The fraction and chlorogenic acid were not cytotoxic. DFT analysis suggest that an electron transfer, followed by proton transfer at carbons 3′OH and 4′OH positions may be the radical scavenging mechanism of chlorogenic acid. Considering this study is bioassay-guided, it is logical to conclude that chlorogenic acid strongly influences the antioxidant capacity of avocado fruit peel.

## 1. Introduction

Oxidative stress remains a culprit in the development and progression of many chronic diseases [1]. It is caused by a disruption in the balance between the biological production of deleterious reactive oxygen species (ROS)/reactive nitrogen species (RNS) and mitigating antioxidant actions [2,3]. ROS and RNS play important roles in redox balance and signaling, immune response, and cellular homeostasis [2]. However, most ROS and RNS are radical species, which are very reactive [3,4]. Thus, their uncontrolled production could lead to oxidative alteration or damage of important biological molecules [1,3,4]. Oxygen, unlike other elements that exists as bi-atomic molecules, has better stability in the triplet state of electron spin (the state of unpaired electrons) than in the singlet state of electron spin (the state of paired electrons), which plays a major role in the reactivity of ROS [2].

ROS and RNS are mostly by-products of biological signaling and/or metabolism. The commonly generated ROS and RNS include hydroxyl radicals, hydrogen peroxide, nitric oxide, peroxides, peroxynitrite, singlet oxygen, and superoxides, which are common by-products of the mitochondrial electron transport, cytochrome P_450_, and NADH oxidase pathways [5]. They have been reported to have detrimental effects on biological molecules, which have been implicated in oxidative stress associated diseases [1,4].

Oxidative stress is a common denominator in the pathology of many chronic diseases (diabetes, cancer, cardiovascular diseases, renal disease, mental and neurological disorders, and pulmonary diseases) and aging due to the oxidative damage caused by ROS and RNS on DNA, proteins, and membrane lipids [1]. Redox signaling is important for proper myocardial functioning and is regulated by several kinases. Oxidative stress can dysregulate this signaling, which can lead to cardiovascular disorders [1]. Oxidative stress can cause oxidative damage to contractile proteins, which may lead to contractile dysfunctions and vascular impairments [6]. ROS may induce pro-tumorigenic signaling, which may promote cancer cell proliferation and survival [7]. In diabetic state, glycation processes can lead to advanced glycation end product (AGE) production [8]. AGEs can induce oxidative stress, which induces several stress-induced transcription factors and pro-inflammatory processes. Lipid peroxidation causes oxidative modifications to biological lipids, including membrane lipids, which compromises cell membrane integrity [1]. Oxidative modifications of lipids, such as apo-lipoprotein B, may lead to formation of oxidized low-density lipoproteins, which has been implicated in the pathology of atherosclerosis [1].

The body is equipped with antioxidant mechanisms to mop deleterious ROS and mitigate the damages caused by oxidative stress [9]. These include antioxidant enzyme systems, such as catalase, superoxide dismutase, and glutathione-related enzyme systems, as well as endogenous antioxidant molecules that quench pro-oxidants or enable the functioning of antioxidant pathways [9]. Dietary or plant-derived polyphenols are known to possess antioxidant capacity, which is key for their medicinal potential in different diseases [10]. Many fruits and vegetables contain these antioxidant phenolics, which contribute to their health benefits [11]. Despite the antioxidant benefits of fruit consumption, studies have shown that the non-edible wastes of many fruits are notable sources of phenolics, which is influential in their reported medicinal properties [12,13,14]. For instance, the seed of avocado fruit has ethnomedicinal applications in mental disorders and obesity [15], while the peel has cosmetic applications [16].

Avocado fruit (*Persea americana* Mill.) is a globally consumed fruit. The cosmetic applications and documented anticancer, antibacterial, anti-inflammatory, and anti-hypertensive potentials of the peel have been strongly linked to its antioxidant properties [16,17]. Phenolic acids, including hydroxybenzoic and hydroxycinnamic acids and flavonoids, including anthocyanins, flavanols, flavanones, and flavonols, are some of the classes of polyphenols reported in the avocado peel, which could contribute to its antioxidant effects [18,19,20]. The radical scavenging activity of the peel extract has been documented [18,19,20,21]. Also, the peel has been shown to exert anti-inflammatory effects in lipopolysaccharide (LPS)-treated murine macrophage cells by suppressing TNF-α expression and NO production [18].

Despite the antioxidant and anti-inflammatory potential of avocado peel, previous studies have not been able to link or correlate its principal phytoconstituents to its bioactivity through comparative experimental analysis, which will give more insight into the antioxidant phytochemistry of the fruit’s peel. In the present study, computational, in vitro, and cell-based experimental models were employed to elucidate the antioxidant phytochemistry of avocado fruit peel.

## 2. Materials and Methods

### 2.1. Procurement of Fruit and Solvent Extraction of Peel

Avocado (*Persea americana* Mill.) fruit (Var. Fuerte) was procured from a local fruit shop. The fruits were washed, and the peel was neatly removed. The peel was air dried and pulverized. Approximately 125 g of the pulverized peel were first defatted with hexane. Thereafter, it was sequentially extracted with dichloromethane, ethyl acetate, methanol, and water, respectively. Extraction was done at a sample-to-solvent ratio of 125 g:1.25 L, respectively, on an orbital shaker [OrbiShake, Model 262, Labotec (Pty) Ltd., Johannesburg, South Africa] set at 125 rpm. For each solvent, extraction was done three times under ambient temperature for 72 h. The organic solvent extracts were recovered by filtering, evaporating [Buchi Rotavapor^®^ R-300, Labotec (Pty) Ltd., Johannesburg, South Africa], and drying under a fume hood. The water extract was recovered by filtering and freeze-drying (Martin Christ Alpha 1–2 LDplus Freeze Dryer, Separations, Johannesburg, South Africa). Tannins were removed from the methanol extract via solid-phase extraction in a polyamide column. All extracts were stored at −20 °C.

### 2.2. Preliminary Phenolic Content and In Vitro Antioxidant Activity Measurements

Total phenol and flavonoid content and Fe^3+^ reducing antioxidant activity (FRAP), as well as 2,2-diphenyl-1-picrylhydrazyl (DPPH), 2,2′-azinobis-(3-ethylbenzothiazoline-6-sulfonic acid) (ABTS), and NO radical scavenging activities were measured in the extracts using methods reported in previous publications. For total phenol and flavonoid content, FRAP, and NO radical scavenging activity, the methods reported by Chukwuma et al. [12] were used. For DPPH and ABTS radical scavenging activities, the methods reported by [22] were used. For the antioxidant assays, ascorbic acid and Trolox were used as the positive controls. For all the above-mentioned assays, the extracts and the positive controls were tested at a concentration of 45 µg/mL.

### 2.3. Fractionation of the Methanol Extract

About 4.1 g of the methanol extract (without tannin) was subjected to a gradient column (silica gel) fractionation. Thin layer chromatography (TLC) was first used to determine the appropriate solvent system for the gradient column fractionation. The mobile phase started with a solvent system volume ratio of 8:1.5:0.5 (hexane, chloroform, and methanol, respectively) and ended with a volume ratio of 0:9:1 (hexane, chloroform, and methanol, respectively). Collected fractions with fairly similar TLC profiles were pooled together into nine different groups labelled as fractions 2 to 10. The fractions were recovered by air drying.

### 2.4. Antioxidant Evaluation of Fractions

Total phenol and flavonoid content, FRAP, and DPPH and ABTS radical scavenging activities were measured for all the 9 fractions and compared to positive controls (ascorbic acid and Trolox). For all the above-mentioned assays, the fractions and positive controls were tested at a concentration of 25 µg/mL.

Fraction 8 (a pool of fractions with similar TLC profile), obtained from a mobile phase solvent system volume ratio of 3:6.5:0.5 (hexane, chloroform, and methanol, respectively), had the highest total phenol content and the most potent FRAP and radical scavenging activity compared to the other fractions. Thus, fraction 8 was subjected to LC-MS analysis.

### 2.5. Liquid Chromatography—Mass Spectroscopic (LC-MS) Quantification

To perform the LC-MS protocol, the following were used: A Waters Synapt G2 (Waters Corporation, Milford, MA, USA), ESI probe, and ESI Pos, at a 15 V cone voltage. The operation of liquid chromatography was done using an Acquity binary solvent manager. The chromatographic column used was a HSS T3 (Waters Corporation, Milford, MA, USA). It had a dimension of 2.1 × 150 mm and a particle size of 1.8 µm. Two solvent systems (A and B) were used as the mobile phase. Solvent system A was water containing 0.1% methanoic acid, while solvent system B was acetonitrile containing 0.1% methanoic acid. A 0.25 mL/min flow rate was applied for the gradient chromatographic separation at 30 °C. The gradient condition followed the following sequence: 100% (A):0% (B), 10 min; 72% (A):28% (B), 21 min; 60% (A):40% (B), 50 s; 0% (A):100% (B), 2 min. The fraction was dissolved in a mixture of acetonitrile and water (1:1 *v*/*v*), passed through a 0.22 µm filter, and injected at a volume of 20 μL. The signals of the separated compounds were recorded at 254 nm. Using the accurate masses, the compounds in the fraction were tentatively determined and semi-quantified (mg/L) relative to rutin standard, and according to the extracted ions.

### 2.6. Dose-Dependent Antioxidant Evaluation of Fraction 8 and Chlorogenic Acid

Dose-dependent in vitro and cell-based models were used to compare the antioxidant effect of fraction 8 with that of chlorogenic acid, which, together with 1-*O*-caffeoylquinic acid (1-CQA), were the most abundant compounds in the fraction. Commercial chlorogenic acid (5-*O*-caffeoylquinic or 5-CQA) purchased form Merck, Johannesburg, South Africa, was used for the assays.

#### 2.6.1. Measurement of Dose-Dependent DPPH Radical Scavenging Activity

The dose-dependent DPPH radical scavenging activity of the samples and positive controls (ascorbic acid and Trolox) were measured at 3.75, 7.5, 15, 30, and 60 µg/mL concentrations.

#### 2.6.2. Measurement of Linoleic Acid Peroxidation Inhibition

The ability of fraction 8 and chlorogenic acid to inhibit FeSO_4_-induced (FS) linoleic acid peroxidation was performed. The detailed protocol for this assay has recently been reported in our publication [23]. In summary, linoleic acid (50 mM) peroxidation was induced using FeSO_4_·7H_2_O (FS) (2 mM in assay volume) after pre-treatment with the different concentrations (5, 10, 20, 40, and 80 µg/mL in assay volume) of the samples and ascorbic acid (positive control). The thiobarbituric acid (TBA) method was used to measure the level of lipid peroxidation. Absorbance readings at 532 nm were used to compute the inhibitory potential of the tested samples on linoleic acid peroxidation as follows:

$$\mathrm{Inhibition}\;\left(\%\right)=\frac{\left(A_{\mathrm{Negative}\;\mathrm{control}}-A_{\mathrm{Normal}\;\mathrm{control}}\right)-(A_{\mathrm{Test}}-A_{\mathrm{Normal}\;\mathrm{control}})}{\left(A_{\mathrm{Negative}\;\mathrm{control}}-A_{\mathrm{Normal}\;\mathrm{control}}\right)}\;\times \;\frac{100}{1}$$

where “A” means “absorbance readings”; the “Normal control” denotes reaction with sample solvent or vehicle, without FS; the “Negative control” denotes reaction with sample solvent or vehicle and FS; and the “Test” denotes reaction with sample and FS.

#### 2.6.3. Measurement of the Antioxidant Capacity in Chang Cells

The effect of the tested samples (fraction 8 and chlorogenic acid) and ascorbic acid (positive control) on lipid peroxidation and the activity of key antioxidant enzymes was measured in Chang liver cells (ATCC^®^ CCL-13™) induced with oxidative stress. The detailed protocol for this assay has recently been reported in our publication [23]. In summary, oxidative stress was induced in cultured cells using FS (1 mM in assay volume) after pre-treatment with the different concentrations (10, 20, 40, and 80 µg/mL in assay volume) of the fraction 8, chlorogenic acid and ascorbic acid. The thiobarbituric acid (TBA) method was used to measure the level of lipid peroxidation in the cell lysate, which was extrapolated from an MDA standard plot as thiobarbituric acid reactive substances (TBARS). Also, the activities of catalase and superoxide dismutase (SOD) were measured in the lysate of cells treated with the highest concentration (80 µg/mL) of the samples using the following assay kits purchased from Merck, Johannesburg, South Africa: Catalase Assay Kit (catalog number: CAT100) and SOD Assay Kit (product number: 19160). The inhibitory potential of the tested samples on lipid peroxidation was computed using the formula below:

$$\mathrm{Inhibition}\;\left(\%\right)=\frac{\left(T_{\mathrm{Negative}\;\mathrm{control}}-T_{\mathrm{Normal}\;\mathrm{control}}\right)-(T_{\mathrm{Test}}-T_{\mathrm{Normal}\;\mathrm{control}})}{\left(T_{\mathrm{Negative}\;\mathrm{control}}-T_{\mathrm{Normal}\;\mathrm{control}}\right)}\;\times \;\frac{100}{1}$$

where “T” is the level of lipid peroxidation in the form of TBARS; the “Normal control” denotes cells treated with the sample solvent/vehicle, without FS; the “Negative control” denotes cells treated with the sample solvent/vehicle and FS; and the “Test” denotes cells treated with the sample and FS.

#### 2.6.4. Measurement of ROS and NO Production Inhibition in RAW 264.7 Cells

The effect of the tested samples (fraction 8 and chlorogenic acid) and ascorbic acid (positive control) on ROS and NO production was measured in RAW 264.7 macrophages [American Type Culture Collection (ATCC) Rockville, MD, USA; ATCC No.: TIB-71 ™] treated with lipopolysaccharide (LPS) (Merck, Johannesburg, South Africa). For ROS and NO production assays, the methods reported by Marrazzo et al. [24] and Njoya et al. [25] were adopted, respectively, with slight modifications.

RAW 264.7 macrophages were grown in Roswell Park Memorial Institute (RPMI) 1640 medium (Separations, Johannesburg, South Africa) supplemented with 10% fetal bovine serum (FBS) and 1% penicillin/streptomycin in a CO_2_ incubator (EC 160, Nϋve; Separations, Johannesburg, South Africa) under standard cell culture conditions (37 °C in a humidified environment with 95% air and 5% CO_2_). At about 80% confluence, the cells were trypsinized with 0.25% trypsin/EDTA (Cytiva Hyclone, USA) and split at a ratio of 1:5 for further passaging. Next, the cytotoxic effect of the tested samples on the RAW 264.7 macrophages was measured at concentrations of (6.25–100 µg/mL) using standard MTT assay protocol.

For the ROS and NO production inhibition assay, confluent RAW 264.7 macrophages were seeded in black/clear-bottom 96-well plate at a density of 10,000 cells per well and incubated for 24 h. The cells were then pre-treated for 2 h with the tested samples. For the ROS production inhibition assay, the cells were treated at sample concentrations of 25, 50, and 100 µg/mL in incubation volume. For the NO production inhibition assay the cells were treated at sample concentrations of 6.25, 12.5, 25, 50, and 100 µg/mL in incubation volume. Some wells were assigned the control and negative control groups, which contained cells treated with the sample solvents/vehicle (≤0.5% DMSO). Next, LPS (200 ng/mL in final incubation volume) was added to the wells containing the samples and the wells assigned as the negative control group, while an equivalent volume of the 0.5% DMSO was added to the wells assigned as the control group. The plates were further incubated for 24 h.

To analyze the cells for ROS production, the cells were washed with phosphate buffered saline (PBS). Next, 100 µL of fresh FBS-free culture medium, containing a 2′,7′dichlorodihydrofluorescein diacetate (DCFH-DA) (Merck, South Africa) fluorescent probe (10 µM in incubation volume), was added into each well with minimal exposure to light, and the plates were further incubated for 30 min. Thereafter, the media containing the fluorescent probe was replaced with PBS, and fluorescence was measured at 485 nm (excitation) and 535 nm (emission) using a SpectraMax iD3 multi-mode microplate reader (Molecular Devices, San Jose, USA). Also, cell images were obtained using a Leica DM IL LED inverted fluorescent microscope (Separations, Johannesburg, South Africa). The inhibitory potential of the tested samples on ROS production was calculated as follows:

$$\mathrm{Inhibition}\;\left(\%\right)=\frac{\left(F_{\mathrm{Negative}\;\mathrm{control}}-F_{\mathrm{Normal}\;\mathrm{control}}\right)-(F_{\mathrm{Test}}-F_{\mathrm{Normal}\;\mathrm{control}})}{\left(F_{\mathrm{Negative}\;\mathrm{control}}-F_{\mathrm{Normal}\;\mathrm{control}}\right)}\;\times \;\frac{100}{1}$$

where “F” means “fluorescence reading”.

To analyze the cells for NO production post-LPS treatment, 100 µL of the incubation medium from each well of the 96-well microtitre plates was aliquoted into designated wells of a new 96-well transparent plate. An equal volume of Griess reagent (Merck, South Africa) was added to the wells and the plate was kept in the dark at room temperature for 15 min. Absorbance values were measured at 540 nm. The concentration of nitrite (µM) was calculated from a sodium nitrite standard linear plot (y = 0.0029x + 0.0114). The inhibitory potential of the tested samples on NO production was calculated as follows:

$$\mathrm{Inhibition}\;\left(\%\right)=\frac{\left(C_{\mathrm{Negative}\;\mathrm{control}}-C_{\mathrm{Normal}\;\mathrm{control}}\right)-(C_{\mathrm{Test}}-C_{\mathrm{Normal}\;\mathrm{control}})}{\left(C_{\mathrm{Negative}\;\mathrm{control}}-C_{\mathrm{Normal}\;\mathrm{control}}\right)}\;\times \;\frac{100}{1}$$

where “C” is the nitrite concentration (μM) extrapolated from the sodium nitrite standard plot.

### 2.7. IC_50_ Computation

The IC_50_ values of the tested samples were computed on GraphPad Prism 7 and/or MS excel 2016 software using the non-linear or linear plot of transformed (log 10) sample concentrations (*x*-axis) versus the corresponding inhibitory bioactivities.

### 2.8. Statistical Analysis

Experimental data was presented as mean ± standard deviation of triplicate analysis (n = 3). The statistical analysis was done on the IBM SPSS Statistics (Windows version 29.0) software using the one-way ANOVA and Tukey post-hoc multiple comparative analysis. Statistical significance was set at *p* < 0.05.

### 2.9. Density Functional Theory (DFT) Analysis

The hydrogen atom transfer (HAT), single electron transfer followed by proton transfer (SET-PT), and sequential proton loss followed by electron transfer (SPLET) mechanisms were used to computationally study the DPPH radical scavenging activity of chlorogenic acid. The equations below sequentially denote the mechanisms for HAT, SET-PT, and SPLET, respectively.

ArOH + R^●^ → ArO^●^ + RH

ArOH + R^●^ → ArOH^●+^ + R^−^ → ArO^●^ + RH

ArOH + R^●^ → ArO^−^ + R^●^ → ArO^●^ + R^−^ → ArO^●^ + RH

where ArO^●^, ArO^−^, ArOH^●+^, R^−^, and RH denote phenolic radical, phenolic anion, phenolic cation, anion radical, and neutral molecule, respectively.

Geometry optimization of all the reaction species was performed using B3LYP together with the 6-311++g (d,p) basis set. This computational method is largely used for radical scavenging mechanisms and has been shown to accurately estimate the geometries and thermodynamic parameters of the reaction species [26]. The bond dissociation enthalpy (BDE), adiabatic ionization potential (AIP), proton dissociation enthalpy (PDE), proton affinity (PA), and electron transfer enthalpy (ETE) (in kcal/mol) of the reaction species for the capture of the DPPH radical by chlorogenic acid (5-CQA) were computed for a methanol-solvent-based reaction, which are parameters that inform the above-mentioned radical reaction mechanism.

## 3. Results

The methanol extract of the fruit peel had the highest (*p* < 0.05) total phenol, while the ethyl acetate extract had the highest (*p* < 0.05) total flavonoid content compared to the other extracts (Table 1). The Fe^3+^ reducing antioxidant activity of the methanol and dichloromethane extracts was significantly (*p* < 0.05) more potent than that of the other extracts and Trolox, but significantly (*p* < 0.05) less potent than that of ascorbic acid (Table 1). The DPPH, ABTS, and NO radical scavenging activity of the methanol extract were stronger than those of the other extracts and in some instances stronger and/or comparable to those of ascorbic acid and Trolox (Table 1).

Among the fractions (fractions 2 to 10) obtained from the methanol extract, fraction 8 consistently showed the highest (*p* < 0.05) total phenol content, as well as DPPH and ABTS radical scavenging and Fe^3+^ reducing antioxidant activity (Table 2). The antioxidant activity of fraction 8 was statistical comparable to that of ascorbic acid and/or Trolox (Table 2).

**Figure 1:**
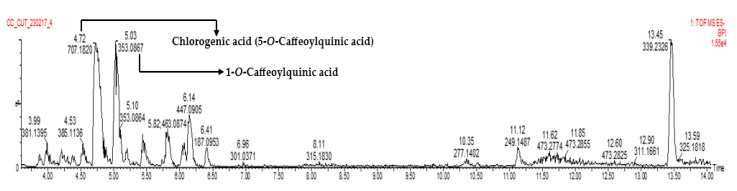
LC-MS chromatogram of fraction 8.

LC-MS analysis of fraction 8 showed the presence of several phenolic compounds, with chlorogenic acid (5-CQA) and 1-*O*-caffeoylquinic acid (1-CQA) being the compounds with the highest concentrations (Table 3, Figure 1, and Supplementary Material). Chlorogenic acid (5-CQA) was the compound of interest in this study.

**Figure 2:**
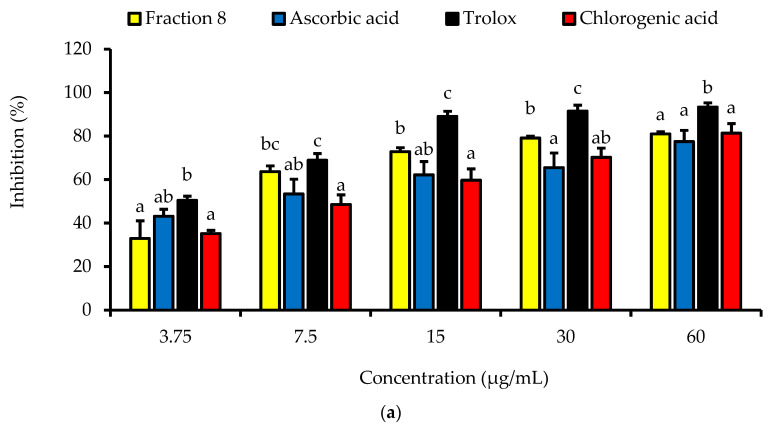

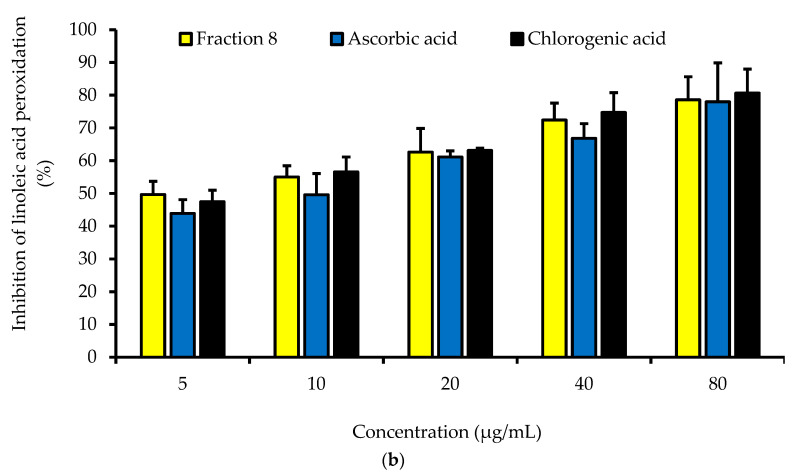
Dose-dependent (**a**) DPPH radical scavenging and (**b**) anti-linoleic acid peroxidative activities of fraction 8 and chlorogenic acid. Data are presented as mean ± SD of triplicate analysis. For each assay, the letters at the top of the bars represent significant differences (*p* < 0.05) between groups when there are no similar letters.

Dose-dependently, the DPPH radical scavenging activity of fraction 8 was statistically (*p* > 0.05) as potent as chlorogenic acid, ascorbic acid, and Trolox (Table 4 and Figure 2a). Although not significantly (*p* > 0.05), the inhibitory effect of fraction 8 (IC_50_ = 5.73 µg/mL) and chlorogenic acid (IC_50_ = 6.17 µg/mL) on FS-induced linoleic acid peroxidation was stronger than that of ascorbic acid (IC_50_ = 8.41 µg/mL) (Table 4 and Figure 2b).

**Figure 3:**
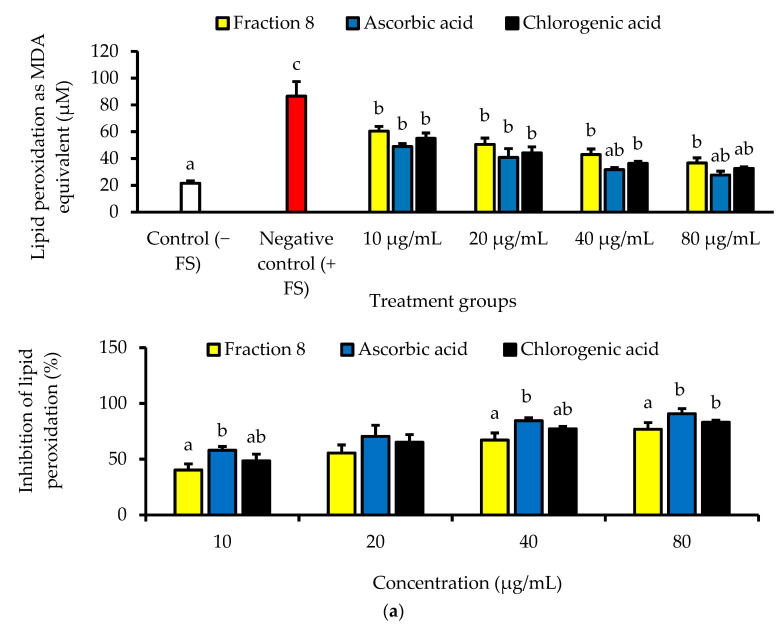

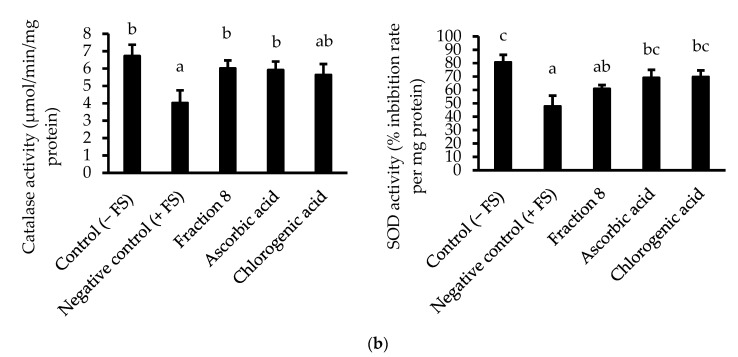
(**a**) Dose-dependent anti-lipid peroxidative and (**b**) antioxidant enzyme modulatory activities of fraction 8 and chlorogenic acid in FS-treated Chang cells. Data are presented as mean ± SD of triplicate analysis. For each assay, statistical comparison was done between the controls (control and negative control) and treatment groups at a given concentration or among the treatment groups at a given concentration. The letters at the top of the bars represent significant differences (*p* < 0.05) between groups when there are no similar letters. “FS” means FeSO4·7H_2_O.

In Chang cells induced with oxidative stress, the anti-lipid peroxidative of ascorbic acid (IC_50_ = 5.74 µg/mL) was significantly (*p* < 0.05) and non-significantly (*p* > 0.05) stronger than that of fraction 8 (IC_50_ = 15.9 µg/mL) and chlorogenic acid (IC_50_ = 9.34 µg/mL), respectively (Table 4 and Figure 3a). Nevertheless, fraction 8, chlorogenic acid, and ascorbic acid (at 80 µg/mL) appreciably mitigated oxidative stress-induced depletion of catalase and SOD enzymes in the Chang cells (Figure 3b).

**Figure 4:**
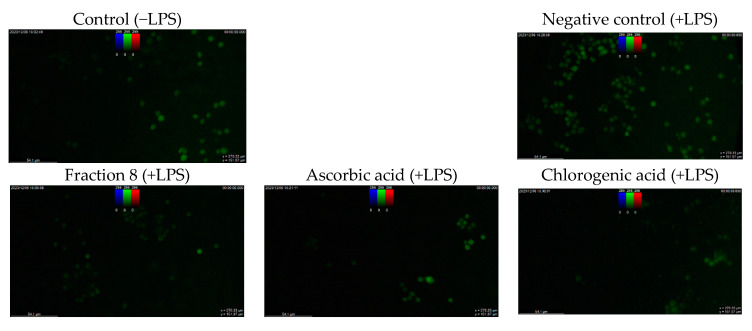

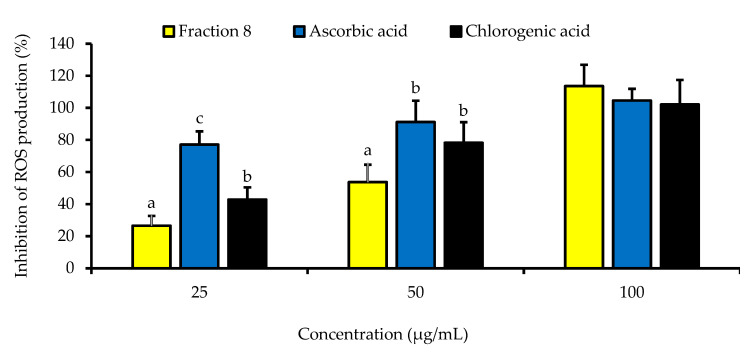
Dose-dependent inhibitory effect of fraction 8 and chlorogenic acid on ROS production in LPS-treated RAW 264.7 macrophages. Data are presented as mean ± SD of triplicate analysis. The letters at the top of the bars represent significant differences (*p* < 0.05) between groups when there are no similar letters. “LPS” means lipopolysaccharide.

**Figure 5:**
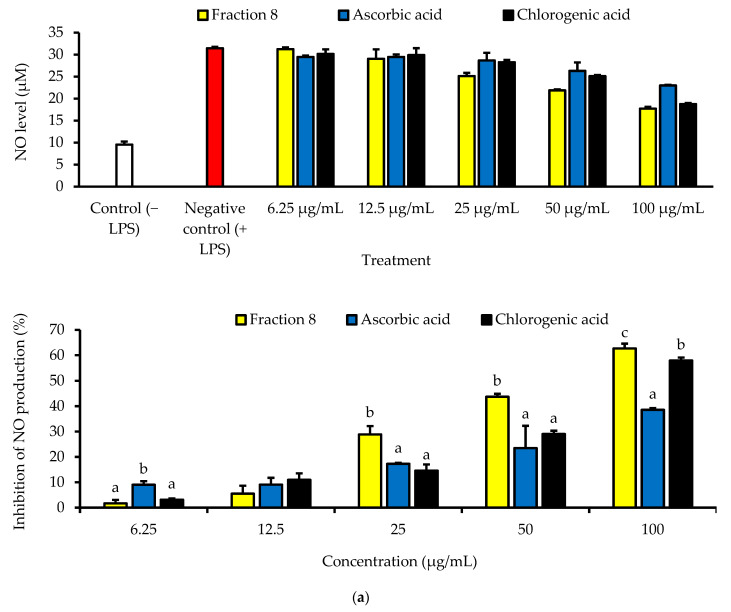

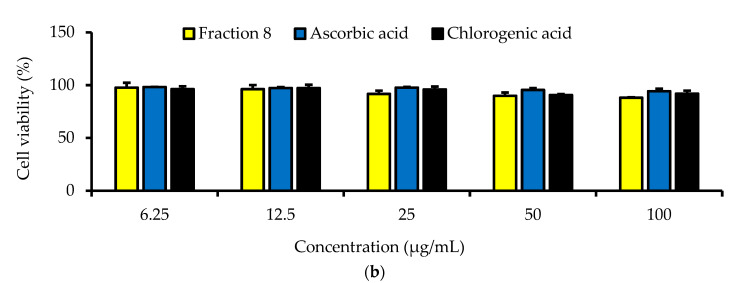
Dose-dependent inhibitory effect of fraction 8 and chlorogenic acid on (**a**) NO production in LPS-treated RAW 264.7 macrophages and (**b**) the viability of RAW 264.7 macrophages. Data are presented as mean ± SD of triplicate analysis. Data are presented as mean ± SD of triplicate analysis. Statistical comparison was done between the controls (control and negative control) and treatment groups at a given concentration or among the treatment groups at a given concentration. The letters at the top of the bars represent significant differences (*p* < 0.05) between groups when there are no similar letters. “LPS” denotes “lipopolysaccharide”.

Although not as potent as ascorbic acid (IC_50_ = 8.08 µg/mL; *p* < 0.05), fraction 8 (IC_50_ = 39.6 µg/mL) and chlorogenic acid (IC_50_ = 28.2 µg/mL) showed promising inhibition of LPS-induced ROS production in RAW 264.7 macrophages (Table 4 and Figure 4). However, the ability of fraction 8 (IC_50_ = 63.5 µg/mL) and chlorogenic acid (IC_50_ = 107 µg/mL) to inhibit NO production in LPS-treated RAW 264.7 macrophages significantly (*p* < 0.05) outperformed that of ascorbic acid (IC_50_ = 203 µg/mL) (Table 4 and Figure 5a). Interestingly, both fraction 8 and chlorogenic acid were not toxic to the RAW 264.7 macrophages (Figure 5b).

**Figure 6:**
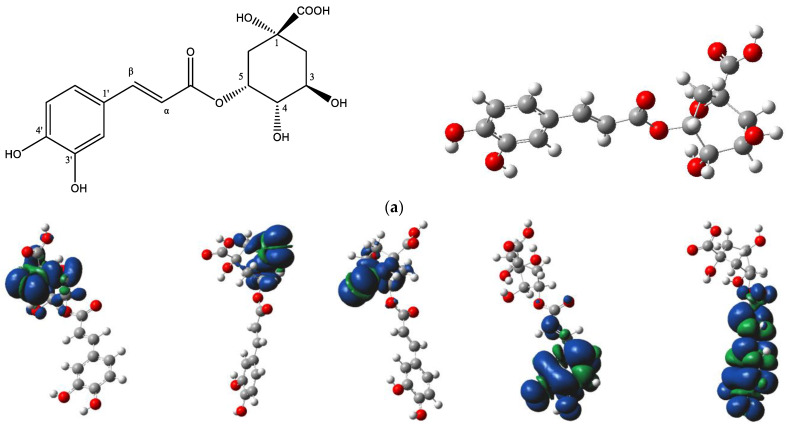

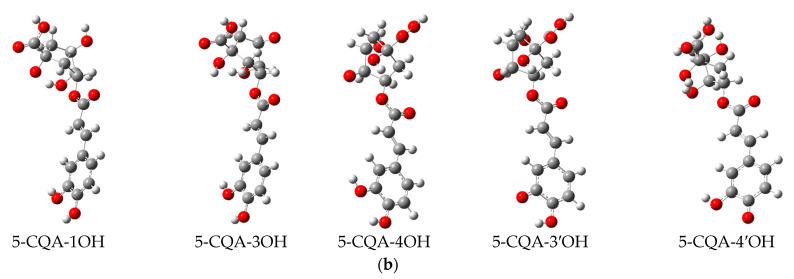
(**a**) Chemical structure of chlorogenic acid (5-*O*-caffeoylquinic acid) and its lowest-energy structural conformer; (**b**) the electron spin density of the optimized structures of 5-*O*-caffeoylquinic acid radicals.

The DFT analysis data are presented in Table 5 and Figure 6. Figure 6a depicts the chemical structure of 5-CQA and the selected hydroxyl group numbering, as well as the lowest energy structural conformer of chlorogenic acid (5-CQA). Figure 6b shows the electron spin density of the optimized structures of 5-CQA radicals for HAT mechanism of DPPH radical scavenging.

For the HAT mechanism, 5-CQA-4′OH H-atom abstraction appears to be the favored mechanism for quenching DPPH radical according to the low BDE value (Table 5) and stable electron distribution on the phenolic radical (Figure 6b). For the SET-PT mechanism, the proton transfer step is more highly favored at positions 3′ and 4′ based on the low PDE values (Table 5). Overall, the SET-PT mechanism at the 5-CQA-3′OH and 5-CQA-4′OH positions appear to be the preferred mechanism for DPPH radical scavenging compared to the HAT and SPLET mechanisms due to the low enthalpy values (Table 5).

## 4. Discussion

Oxidative stress is pivotal in the pathological outcomes of many diseases, which has been linked to the oxidative damage caused by deleterious ROS on biological molecules [1]. The peel of avocado fruit has been shown to have antioxidant effects [18,19,20]. In this study, we presented the antioxidant phytochemistry of avocado fruit peel.

The highly reactive nature of biological radicals notably influences their ability to oxidatively damage biomolecules. Plant polyphenols, on the other hand, are known radical scavengers, due to their ability to transfer hydroxyl H-atom and form stable phenoxy radicals [27]. Also, the iron-chelating ability of polyphenols contributes to their antioxidant potential [28]. In the present study, the methanol extract of avocado fruit peel had the highest polyphenol content and consistently had the highest radical scavenging and Fe^3+^ reducing activity relative to the other extracts (Table 1). Although not on the same variety, previous studies have documented the potent radical scavenging effects of the peel’s methanol extract [21,29]. Consistent with our study, the methanol extract was more potent than ethyl acetate extract [29]. Among all the fractions form the methanol extract, fraction 8 had the most potent radical scavenging and Fe^3+^ reducing activity (Table 2). The activity correlates with the total phenol content (Table 2), confirming the influence of polyphenols on the radical quenching potential of plants. Supporting documented evidence has also shown correlations between the phenolic contents of avocado peel extract and its radical scavenging and in vitro antioxidant capacity [20,29]. In fact, relative to the pulp and seed, studies have shown that the peel possessed more phenolic content, and thus had stronger radical scavenging and antioxidant capacity [20,30,31].

Previous studies have reported the presence of phenolic acids, including hydroxybenzoic and hydroxycinnamic acids and flavonoids, including anthocyanins, flavanols, flavanones, and flavonols in avocado peel [18,19,20], which were partly identified in fraction 8 (Table 3). Also, the phenol content of fraction 8 was higher than the flavonoid content (Table 2), which suggests phenolic compounds may have influenced its antioxidant capacity more than the flavonoids. A consistent trend of data has previously been reported in fractions obtained from the methanol and ethyl acetate extracts of peel of an avocado variety grown in Ampelgading, Indonisa [28]. It was shown that the phenolic content contributed more to the radical scavenging and ferric reducing activity of the fractions than the flavonoids content [28]. In the study, 1,2,4-trihidroksiheptadek-12,16-diyne and 1,2,4-trihidroksiheptadek-16-yne-18-ene were identified as the possible antioxidant compounds in the fraction obtained from the methanol and ethyl acetate extracts, respectively [28]. However, this study [28], like other studies [18,19,20], did not compare the antioxidant activity of the identified compounds with that of the potent extracts or fractions in which they were identified. Thus, the study was not able to ascertain whether the identified compounds influence the antioxidant capacity of the peel extracts or fractions. This suggests a gap in these studies and provides the rationale and relevance of our study.

In our study, there was a predominant presence of chlorogenic and 1-*O*-caffeoylquinic acid in fraction 8 (Table 3). Chlorogenic acid and derivatives have been reported in avocado peel [Rodríguez-Martínez; Hirasawa]. The data of our study further showed that the radical scavenging activity chlorogenic acid was comparable to that of fraction 8 and ascorbic acid (Table 4 and Figure 2a), suggesting that chlorogenic acid may strongly influence the antioxidant effect of fraction 8. Moreover, chlorogenic acid is a known dietary phenolic acid with antioxidant and anti-inflammatory potentials [32,33,34]. Further computational DFT analysis suggests that the favored radical scavenging mechanism of chlorogenic acid is a single electron transfer followed by proton transfer at 3′OH and 4′OH positions (Table 5 and Figure 6).

Furthermore, the radical scavenging potency of fraction 8 and chlorogenic acid (Table 4) may be relevant in their anti-lipid peroxidative attributes. This is because during lipid peroxidation free radicals can oxidatively attack biological lipids, including membrane lipids, compromising cell integrity, and exacerbating oxidative stress [35]. In vitro and in Chang cells, fraction 8 and chlorogenic acid dose-dependently inhibited lipid peroxidation (Table 4 and Figure 2b and Figure 3a). In Chang cells, the anti-lipid peroxidative effect of fraction 8 and chlorogenic acid was accompanied by the modulation of SOD and catalase activities (Figure 3b), which are key antioxidants enzymes. The anti-lipid peroxidative and antioxidant enzyme modulatory effect of chlorogenic acid was comparable to that of ascorbic acid and fraction 8, suggesting chlorogenic acid may notably influence the anti-lipid peroxidative effect of fraction 8. Moreover, the anti-lipid peroxidative and antioxidant enzyme modulatory effect of chlorogenic acid has been documented in animal disease models [36,37].

In LPS-treated RAW 264.7 macrophages, fraction 8 and chlorogenic acid dose-dependently suppressed oxidative stress by suppressing ROS production (Table 4 and Figure 4). This data, together with the potent anti-lipid peroxidative and antioxidant enzyme modulatory action of chlorogenic acid (Figure 3) strongly supports the documented modulatory action of chlorogenic acid on biological redox status [32,33,36,37], and further supports its notable contribution to the antioxidant properties of avocado peel. Furthermore, both fraction 8 and chlorogenic acid dose-dependently suppressed NO production in LPS-treated RAW 264.7 macrophages (Table 4 and Figure 5a) without causing cytotoxicity (Figure 5b). In fact, they outperformed ascorbic acid. Immune myeloid cells, like macrophages, are known to produce more cytokines and NO in response to inflammatory signals [38]. Thus, the inhibitory action of fraction 8 on NO production in RAW 264.7 macrophages induced with inflammation suggests an anti-inflammatory potential. Through the suppressive action on LPS-induced NO production and TNF-α expression in RAW 264.7 macrophages, existing data have shown the anti-inflammatory action of the hydroalcoholic extract of avocado peel [18]. Data of our study further shows that the anti-inflammatory potential of avocado peel may be strongly influenced by the presence of chlorogenic acid, considering the promising suppressive effect of chlorogenic acid on LPS-induced NO production (Table 4 and Figure 5a). Moreover, chlorogenic acid has been reported to potentiate anti-inflammatory action by moderating the production and action of inflammatory mediators, including TNF-α, IL-1β, IL-6, IL-8, NO, and PGE2, while modulating signaling processes associated with NF-κB, MAPK, Nrf2, etc. [34].

## 5. Conclusions

In this study, a bioassay-guided approach was used to elucidate the antioxidant phytochemistry of avocado peel. Fractionation of the peel’s methanol extract yielded a fraction that had promising ability to scavenge free radical, inhibit lipid peroxidation, suppress ROS and NO production and modulate antioxidant enzyme activity. The fraction also showed the predominant presence of chlorogenic acid. Data further showed that chlorogenic acid had antioxidant activities that were comparable to that of the fraction and ascorbic acid. It scavenged DPPH radical through a single electron transfer, followed by proton transfer at its carbons 3′OH and 4′OH positions. Considering that our investigation was bioassay-guided, it is logical to conclude that chlorogenic acid is a bioactive principle that notably influences the antioxidant capacity of avocado fruit peel.

## Figures and Tables

**Table 1:** Total phenol and flavonoid contents and in vitro Fe^3+^ reducing and radical scavenging activity of crude extract at 45 µg/mL.

Groups	Total Phenol Content (mg/g GAE)	Total Flavonoid Content (mg/g QE)	Fe^3+^ Antioxidant Capacity (mg/g GAE)	DPPH Radical Scavenging Activity (%)	ABTS Radical Scavenging Activity (%)	NO Radical Scavenging Activity (%)
DCM	612 ± 48.2 ^b^	6.94 ± 0.76 ^a^	163 ± 0.61 ^b^	68.1 ± 5.80 ^b^	55.0 ± 5.31 ^b^	52.9 ± 2.26 ^d^
EtAc	142 ± 3.60 ^a^	15.8 ± 2.24 ^b^	31.8 ± 1.95 ^a^	29.1 ± 1.26 ^a^	31.8 ± 3.98 ^a^	33.4 ± 4.78 ^c^
MeOH	829 ± 48.6 ^c^	8.17 ± 0.60 ^a^	162 ± 0.42 ^b^	93.9 ± 2.79 ^c^	96.3 ± 3.14 ^c^	62.6 ± 3.03 ^d^
Water	157 ± 2.62 ^a^	7.49 ± 0.19 ^a^	38.7 ± 0.16 ^a^	35.2 ± 1.80 ^a^	40.8 ± 0,96 ^a^	29.8 ± 6.51 ^bc^
Asc	N/A	N/A	253 ± 28.1 ^c^	87.5 ± 5.86 ^c^	99.8 ± 0.16 ^c^	11.1 ± 3.46 ^a^
Trolox	N/A	N/A	57.1 ± 4.77 ^a^	94.6 ± 1.25 ^c^	98.1 ± 3.10 ^c^	20.0 ± 2.21 ^ab^

“ABTS”, 2,2′-azino-bis(3-ethylbenzothiazoline-6-sulfonic acid); “Asc”, ascorbic acid; “DCM”, dichloromethane extract; “DPPH”, 2,2-diphenyl-1-picrylhydrazyl; “EtAc”, ethyl acetate extract; “GAE”, gallic acid equivalent; “MeOH”, methanol extract; “N/A”, not applicable; “NO”, nitric oxide; “QE”, quercetin equivalent. Data are presented as mean ± SD of triplicate analysis. For each assay, the superscript letters represent significant differences (*p* < 0.05) between groups when there are no similar letters.

**Table 2:** Total phenol and flavonoid contents and in vitro Fe^3+^ reducing and radical scavenging activity of crude extract at 25 µg/mL.

Groups	Total Phenol Content (mg/g GAE)	Total Flavonoid Content (mg/g QE)	DPPH Radical Scavenging (%)	ABTS Radical Scavenging (%)	Fe^3+^ Antioxidant Capacity (mg/g GAE)
F2	195 ± 1.42 ^ab^	17.1 ± 4.00 ^ab^	44.2 ± 1.57 ^bc^	13.7 ± 0.21 ^b^	24.8 ± 3.75 ^ab^
F3	238 ± 12.5 ^bc^	47.3 ± 4.10 ^cd^	55.6 ± 1.03 ^c^	40.9 ± 3.03 ^f^	45.2 ± 2.52 ^b^
F4	175 ± 4.21 ^a^	84.45 ± 12.4 ^e^	40.8 ± 1.37 ^ab^	44.5 ± 0.03 ^f^	46.4 ± 0.51 ^b^
F5	236 ± 10.6 ^bc^	8.04 ± 3.05 ^a^	32.5 ± 5.6 ^a^	30.1 ± 0.65 ^e^	29.6 ± 0.51 ^ab^
F6	252 ± 10.1 ^cd^	29.3 ± 8.10 ^bc^	43.2 ± 5.64 ^ab^	22.5 ± 1.53 ^d^	30.6 ± 6.49 ^ab^
F7	339 ± 3.60 ^e^	63.7 ± 14.9 ^de^	68.8 ± 1.26 ^d^	6.57 ± 0.01 ^a^	25.6 ± 1.26 ^ab^
F8	519 ± 48.6 ^f^	39.4 ± 5.28 ^bc^	90.8 ± 9.10 ^e^	89.4 ± 4.65 ^g^	96.7 ± 15.9 ^c^
F9	288 ± 11.2 ^d^	7.00 ± 0.40 ^a^	51.9 ± 2.87 ^bc^	15.4 ± 0.65 ^bc^	19.0 ± 2.83 ^a^
F10	179 ± 18.7 ^a^	23.6 ±7.47 ^ab^	44.7 ± 0.33 ^b^	20.6 ± 1.32 ^cd^	44.3 ± 17.7 ^b^
Asc	N/A	N/A	85.5 ± 1.32 ^e^	99.4 ± 0.15 ^h^	ND
Trolox	N/A	N/A	90.5 ± 2.52 ^e^	99.9 ± 0.06 ^h^	89.1 ± 13.0 ^c^

“ABTS”, 2,2′-azino-bis(3-ethylbenzothiazoline-6-sulfonic acid); “Asc”, ascorbic acid; “DPPH”, 2,2-diphenyl-1-picrylhydrazyl; “F2” to “F10”, fraction 2 to fraction 10; “GAE”, gallic acid equivalent; “N/A”, not applicable; “ND”, not determined; “QE”, quercetin equivalent. Data are presented as mean ± SD of triplicate analysis. For each assay, the superscript letters represent significant differences (*p* < 0.05) between groups when there are no similar letters.

**Table 3:** LC-MS data of fraction 8.

Identified Compounds	Ontology	ART (min)	AMz	Average Conc. (mg/L)
4-Hydroxy-3-prenylbenzoic acid glucoside	Phenolic glycosides	3.176	413.14206	0.1
Microperfuranone	Butenolides	3.423	265.08621	0.5
Eudesmic acid	Gallic acid and derivatives	4.359	211.05984	0.1
Butalactin	Gamma butyrolactones	4.554	241.07141	0.1
Chlorogenic acid	Quinic acids and derivatives	4.72	353.08762	103.5
4-Hydroxybenzoic acid	Hydroxybenzoic acid derivatives	4.917	137.02409	0.1
Mycophenolic acid	Phthalides	5.014	319.12024	0.1
1-*O*-caffeoylquinic acid	Quinic acids and derivatives	5.029	353.0867	102.3
Caffeic acid	Hydroxycinnamic acids	5.164	179.0349	1.0
Quercetin 3-lathyroside	Flavonoid-3-O-glycosides	5.445	595.1261	21.9
Rutin	Flavonoid-3-O-glycosides	5.671	609.14667	1.0
Quercetin 3-galactoside	Flavonoid-3-O-glycosides	5.816	463.08737	30.0
Crepidiaside A	O-glycosyl compounds	5.868	421.14926	0.1
Gibberellin A17	C20-gibberellin 20-carboxylic acids	6.048	377.16031	0.1
Quercetin 3-[rhamnosyl-(1->2)-alpha-L-arabinopyranoside]	Flavonoid-3-O-glycosides	6.055	579.13745	14.2
Quercitrin	Flavonoid-3-O-glycosides	6.145	447.09045	43.4
Azelaic acid	Medium-chain fatty acids	6.41	187.09761	16.2
3-[4-[1-(4-Hydroxy-3-methoxyphenyl)-1.3-dihydroxyisopropoxy]-2-hydroxyphenyl]propyl alpha-L-rhamnopyranoside	Lignan glycosides	6.426	509.20142	0.1
Quercetin	Flavonols	6.978	301.03497	3.0
Corchorifatty acid F	Lineolic acids and derivatives	7.748	327.21799	0.1
Acrostalidic acid	Naphthopyrans	10.332	277.1442	5.4
Kopetdaghin A	Sesquiterpenoids	10.488	383.22238	0.5
Xanthoxin	Sesquiterpenoids	11.134	249.14981	16.5
etretinate	Retinoid esters	11.38	353.21255	4.2
3-Oxo-4.6-choladienoic acid	Bile acids. alcohols and derivatives	12.203	369.24011	3.4
1.1′-[1.11-Undecanediylbis(oxy)]bisbenzene	Phenol ethers	13.462	339.23151	108.8
Canrenone	Steroid lactones	14.821	339.19983	10.9

ART means “average retention time”; AMz means “average mass-to-charge ratio”; Conc. means “concentration”.

**Table 4:** IC_50_ values for the dose-dependent activities of the tested samples.

Assays	Fraction 8	Ascorbic Acid	Trolox	Chlorogenic Acid
	IC_50_ Values (µg/mL)			
DPPH radical scavenging	5.59 ± 1.75 ^ab^	6.50 ± 2.18 ^ab^	2.41 ± 0.46 ^b^	8.84 ± 1.44 ^a^
Anti-linoleic acid peroxidation	5.73 ± 1.10	8.41 ± 3.33	ND	6.17 ± 1.26
Anti-lipid peroxidation in Chang liver cells	15.9 ± 3.16 ^a^	5.74 ± 0.66 ^b^	ND	9.34 ± 2.40 ^b^
Inhibition of ROS production in RAW 264.7 cells	39.6 ± 3.05 ^a^	8.08 ± 1.83 ^c^	ND	28.2 ± 2.50 ^b^
Inhibition of NO production in RAW 264.7 cells	63.5 ± 4.89 ^c^	203 ± 26.8 ^a^	ND	107 ± 2.06 ^b^

“ND” means “not determined”. Data are presented as mean ± SD of triplicate analysis. For each assay, the superscript letters attached to each data represent significant differences (*p* < 0.05) between groups when there are no similar letters.

**Table 5:** Bond dissociation enthalpy (BDE), adiabatic ionization potential (AIP), proton dissociation enthalpy (PDE), proton affinity (PA), and electron transfer enthalpy (ETE) obtained (in kcal/mol) of the reaction species for the capture of the DPPH radical via 5-CQA.

Structure *	HAT	SET-PT		SPLET	
	BDE	AIP	PDE	PA	ETE
	kcal/mol				
5-CQA-1OH	27.6	22.0	5.6	14.0	13.6
5-CQA-3OH	24.7	22.0	2.6	22.1	2.6
5-CQA-4OH	27.1	22.0	5.0	24.1	3.0
5-CQA-3′OH	2.6	22.0	−19.4	5.0	−2.4
5-CQA-4′OH	2.1	22.0	−19.9	5.3	−3.2

* The numbers 1, 3, 4, 3′, and 4’ denote the position of the eliminated hydrogen from 5-CQA.

## Data Availability

The data of this study is available from the corresponding author on request.

## Supplementary Materials

The following supporting information can be downloaded at: https://www.mdpi.com/article/10.3390/antiox13040456/s1.
